# Arrangements of Approaching Pseudo-Lines

**DOI:** 10.1007/s00454-021-00361-w

**Published:** 2022-01-22

**Authors:** Stefan Felsner, Alexander Pilz, Patrick Schnider

**Affiliations:** 1 Institut für Mathematik, Technische Universität, Berlin, Germany; 2 Institute of SoftwareTechnology, Graz University of Technology, Graz, Austria; 3 Department of Computer Science, ETH Zürich, Zürich, Switzerland

**Keywords:** Pseudo-line arrangements, Order types, Discrete geometry, 52C30

## Abstract

We consider arrangements of *n* pseudo-lines in the Euclidean plane where each pseudo-line $\ell _i$ is represented by a bi-infinite connected *x*-monotone curve $f_i(x)$, $x \in \mathbb {R}$, such that for any two pseudo-lines $\ell _i$ and $\ell _j$ with $i \!<\! j$, the function $x \!\mapsto \! f_j(x) \!-\! f_i(x)$ is monotonically decreasing and surjective (i.e., the pseudo-lines approach each other until they cross, and then move away from each other). We show that such *arrangements of approaching pseudo-lines*, under some aspects, behave similar to arrangements of lines, while for other aspects, they share the freedom of general pseudo-line arrangements. For the former, we prove:

- There are arrangements of pseudo-lines that are not realizable with approaching pseudo-lines.
- Every arrangement of approaching pseudo-lines has a dual generalized configuration of points with an underlying arrangement of approaching pseudo-lines.

For the latter, we show:

- There are $2^{\Theta (n^2)}$ isomorphism classes of arrangements of approaching pseudo-lines (while there are only $2^{\Theta (n \log n)}$ isomorphism classes of line arrangements).
- It can be decided in polynomial time whether an allowable sequence is realizable by an arrangement of approaching pseudo-lines.

Furthermore, arrangements of approaching pseudo-lines can be transformed into each other by flipping triangular cells, i.e., they have a connected flip graph, and every bichromatic arrangement of this type contains a bichromatic triangular cell.

There are $2^{\Theta (n^2)}$ isomorphism classes of arrangements of approaching pseudo-lines (while there are only $2^{\Theta (n \log n)}$ isomorphism classes of line arrangements).

## Introduction

Arrangements of lines and, in general, arrangements of hyperplanes are paramount data structures in computational geometry whose combinatorial properties have been extensively studied, partially motivated by the point-hyperplane duality. Pseudo-line arrangements are a combinatorial generalization of line arrangements. Defined by Levi in 1926 the full potential of working with these structures was first exploited by Goodman and Pollack.

While pseudo-lines can be considered either as combinatorial or geometric objects, they also lack certain geometric properties that may be needed in proofs. The following example motivated the research presented in this paper.

**Fig. 1:**
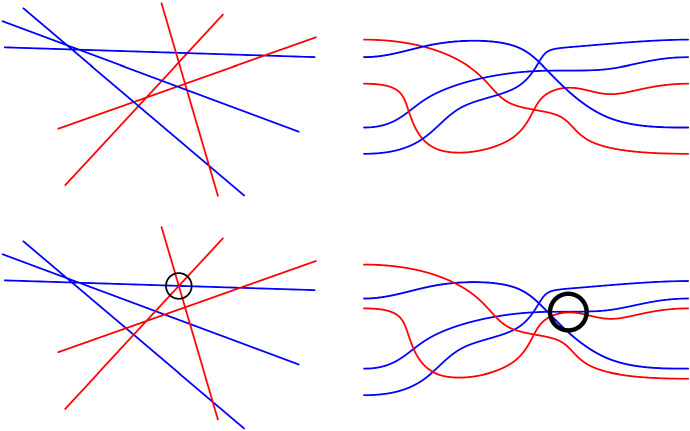
Vertical translation of the red lines shows that there is always a bichromatic triangle in a bichromatic line arrangement (left). For pseudo-line arrangements, a vertical translation may result in a structure that is no longer a valid pseudo-line arrangement (right)

Consider a finite set of lines that are either red or blue, no two of them parallel and no three of them passing through the same point. Every such arrangement has a bichromatic triangle, i.e., an empty triangular cell bounded by red and blue lines. This can be shown using a distance argument similar to Kelly’s proof of the Sylvester–Gallai theorem (see, e.g., [2, p. 73]). We sketch another nice proof. Think of the arrangement as a union of two monochromatic arrangements in colors blue and red. Continuously translate the red arrangement in positive *y*-direction while keeping the blue arrangement in place. Eventually the combinatorics of the union arrangement will change with a triangle flip, i.e., with a crossing passing a line. The area of monochromatic triangles is not affected by the motion. Therefore, the first triangle that flips is a bichromatic triangle in the original arrangement. See Fig. 1 (left).

This argument does not generalize to pseudo-line arrangements. See Fig. 1 (right). Actually the question whether all simple bichromatic pseudo-line arrangements have bichromatic triangles is by now open for several years. The crucial property of lines used in the above argument is that shifting a subset of the lines vertically again yields an arrangement, i.e., the shift does not introduce multiple crossings. We were wondering whether any pseudo-line arrangement can be drawn such that this property holds. In this paper, we show that this is not true and that arrangements where this is possible constitute an interesting class of pseudo-line arrangements.

Define an *arrangement of pseudo-lines* as a finite family of *x*-monotone bi-infinite connected curves (called *pseudo-lines*) in the Euclidean plane such that each pair of pseudo-lines intersects in exactly one point, at which they cross. For simplicity, we consider the *n* pseudo-lines $\{\ell _1, \ldots , \ell _n\}$ to be indexed from 1 to *n* in top-bottom order at left infinity.^1^ A pseudo-line arrangement is *simple* if no three pseudo-lines meet in one point; if in addition no two pairs of pseudo-lines cross at the same *x*-coordinate we call it *x*-*simple.*

An *arrangement of approaching pseudo-lines* is an arrangement of pseudo-lines where each pseudo-line $\ell _i$ is represented by function-graph $f_i(x)$, defined for all $x \in \mathbb {R}$, such that for any two pseudo-lines $\ell _i$ and $\ell _j$ with $i < j$, the function $x \mapsto f_i(x) - f_j(x)$ is monotonically decreasing and surjective. This implies that the pseudo-lines approach each other until they cross, and then they move away from each other, and exactly captures our objective to vertically translate pseudo-lines in an arbitrary way while maintaining the invariant that the collection of curves is a valid pseudo-line arrangement (if $f_i-f_j$ is not surjective the crossing of pseudo-lines *i* and *j* may disappear upon vertical translations). For most of our results, we consider the pseudo-lines to be *strictly approaching*, i.e., the function is strictly decreasing. For simplicity, we may sloppily call arrangements of approaching pseudo-lines *approaching arrangements*.

In this paper, we identify various notable properties of approaching arrangements. In Sect. 2, we show how to modify approaching arrangements and how to decide whether an arrangement is *x*-isomorphic^2^ to an approaching arrangement in polynomial time. Then, we show a specialization of Levi’s enlargement lemma for approaching pseudo-lines and use it to show that arrangements of approaching pseudo-lines are dual to generalized configurations of points with an underlying arrangement of approaching pseudo-lines. In Sect. 5, we describe arrangements which have no realization as approaching arrangement. We also show that asymptotically there are as many approaching arrangements as pseudo-line arrangements. We conclude in Sect. 6 with a generalization of the notion of being approaching to three dimensions; it turns out that arrangements of approaching pseudo-planes are characterized by the combinatorial structure of the family of their normal vectors at all points.

**Related work** Restricted representations of Euclidean pseudo-line arrangements have been considered already in early work about pseudo-line arrangements. Goodman [8] shows that every arrangement has a representation as a *wiring diagram*. More recently there have been results on drawing arrangements as convex polygonal chains with few bends [6] and on small grids [5]. Goodman and Pollack [11] consider arrangements whose pseudo-lines are the function-graphs of polynomial functions with bounded degree. In particular, they give bounds on the degree necessary to represent all isomorphism classes of pseudo-line arrangements. Generalizing the setting to higher dimensions (by requiring that any pseudo-hyperplane can be translated vertically while maintaining that the family of hyperplanes is an arrangement) we found that such approaching arrangements are representations of *Euclidean oriented matroids*, which are studied in the context of pivot rules for oriented matroid programming (see [4, Chap. 10]).

## Manipulating Approaching Arrangements

Lemma 2.1 shows that we can make the pseudo-lines of approaching arrangements piecewise linear. This is similar to the transformation of Euclidean pseudo-line arrangements to equivalent wiring diagrams. Before stating the lemma it is appropriate to briefly discuss notions of isomorphism for arrangements of pseudo-lines.

Since we have defined pseudo-lines as *x*-monotone curves there are two faces of the arrangement containing the points at ± infinity of vertical lines. These two faces are the *north-face* and the *south-face*. A *marked arrangement* is an arrangement together with a distinguished unbounded face, the north-face. Pseudo-lines of marked arrangements are oriented such that the north-face is to the left of the pseudo-line. We think of pseudo-line arrangements and in particular of approaching arrangements as being marked arrangements.

Two pseudo-line arrangements are *isomorphic* if there is an isomorphism of the induced cell complexes which maps north-face to north-face and respects the induced orientation of the pseudo-lines. Two pseudo-line arrangements are *x-isomorphic* if a sweep with a vertical line meets the crossings in the same order.

Both notions can be described in terms of allowable sequences. An *allowable sequence* is a sequence of permutations starting with the identity permutation $\mathrm{id}= (1, \ldots , n)$ in which (i) a permutation is obtained from the previous one by the reversal of one or more non-overlapping substrings, and (ii) each pair is reversed exactly once. An allowable sequence is *simple* if two adjacent permutations differ by the reversal of exactly two adjacent elements.

Note that the permutations in which a vertical sweep line intersects the pseudo-lines of an arrangement gives an allowable sequence. We refer to this as *the allowable sequence* of the arrangement and say that the arrangement *realizes* the allowable sequence. Clearly two arrangements are *x*-isomorphic if they realize the same allowable sequence.

Replacing the vertical line for the sweep by a moving curve (vertical pseudo-line) which joins north-face and south-face and intersects each pseudo-line of the arrangement exactly once we get a notion of pseudo-sweep. A pseudo-sweep typically has various options for making progress, i.e., for passing a crossing of the arrangement. Each pseudo-sweep also produces an allowable sequence. Two arrangements are isomorphic if their pseudo-sweeps yield the same collection of allowable sequences or equivalently if there are pseudo-sweeps on the two arrangements which produce the same allowable sequence.

### Lemma 2.1

For any arrangement of approaching pseudo-lines, there is an *x*-isomorphic arrangement of approaching polygonal curves (starting and ending with a ray). If the allowable sequence of the arrangement is simple, then there exists such an arrangement without crossings at the bends of the polygonal curves.

### Proof

**Fig. 2:**
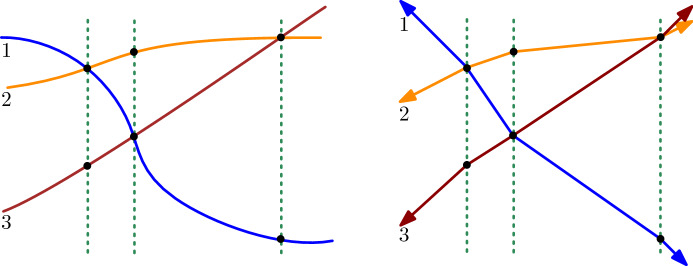
Transforming an arrangement of approaching pseudo-lines into an isomorphic one of approaching polygonal pseudo-lines

Consider the approaching pseudo-lines and add a vertical ‘helper-line’ at every crossing. Connect the intersection points of each pseudo-line with adjacent helper-lines by segments. This results in an arrangement of polygonal curves between the leftmost and the rightmost helper-line. See Fig. 2. Since the original pseudo-lines were approaching, these curves are approaching as well; the signed distance between the intersection points with the vertical lines is decreasing, and this property is maintained by the linear interpolations between the points. To complete the construction, we add rays in negative *x*-direction starting at the intersection points at the first-helper line; the slopes of the rays are to be chosen such that their order reflects the order of the original pseudo-lines at left infinity. After applying the analogous construction at the rightmost helper-line, we obtain the *x*-isomorphic arrangement. If the allowable sequence of the arrangement is simple, we may choose the helper-lines between the crossings and use a corresponding construction. This avoids an incidence of a bend with a crossing. $\square$

The construction used in the proof yields pseudo-lines being represented by polygonal curves with a quadratic number of bends. It might be interesting to consider the problem of minimizing bends in such polygonal representations of arrangements. Two simple operations which can help to reduce the number of bends are *horizontal stretching*, i.e., a change of the *x*-coordinates of the helper-lines which preserves their left-to-right order, and *vertical shifts* which can be applied a helper-line and all the points on it. Both operations preserve the *x*-isomorphism class.

The two operations are crucial for our next result, where we show that the intersection points with the helper-lines can be obtained by a linear program. Asinowski [3] defines a *suballowable sequence* as a sequence obtained from an allowable sequence by removing an arbitrary number of permutations from it. An arrangement thus realizes a suballowable sequence if we can obtain this suballowable sequence from its allowable sequence.

### Theorem 2.2

Given a suballowable sequence, we can decide in polynomial time whether there is an arrangement of approaching pseudo-lines with such a sequence.

### Proof

We attempt to construct a polygonal pseudo-line arrangement for the given suballowable sequence. As discussed in the proof of Lemma 2.1, we only need to obtain the points in which the pseudo-lines intersect vertical helper-lines through crossings. The allowable sequence of the arrangement is exactly the description of the relative positions of these points. We can consider the *y*-coordinates of pseudo-line $\ell _i$ at a vertical helper-line $v_c$ as a variable $y_{i,c}$ and by this encode the suballowable sequence as a set of linear inequalities on those variables, e.g., to express that $\ell _i$ is above $\ell _j$ at $v_c$ we use the inequality $y_{i,c} \ge y_{j,c} +1$. Further, the curves are approaching if and only if $y_{i, c} - y_{j,c} \ge y_{i,c+1} - y_{j, c+1}$ for all $1\le i<j \le n$ and *c*. These constraints yield a polyhedron (linear program) that is non-empty (feasible) if and only if there exists such an arrangement. Since the allowable sequence of an arrangement of *n* pseudo-lines consists of $$\left( {\begin{array}{c}n\\ 2\end{array}}\right) +1$$ permutations the linear program has $O(n^4)$ inequalities in $O(n^3)$ variables. Note that it is actually sufficient to have constraints only for neighboring points along the helper lines, this shows that $O(n^3)$ inequalities are sufficient. $\square$

Let us emphasize that deciding whether an allowable sequence is realizable by a line arrangement is an $\exists \mathbb {R}$-hard problem [15], and thus not even known to be in NP. While we do not have a polynomial-time algorithm for deciding whether there is an isomorphic approaching arrangement for a given pseudo-line arrangement^3^, Theorem 2.2 tells us that the problem is in NP, as we can give the order of the crossings encountered by a sweep as a certificate for a realization. The corresponding problem for lines is also $\exists \mathbb {R}$-hard [18].

The following observation is the main property that makes approaching pseudo-lines interesting.

### Observation 2.3

Given an arrangement *A* of strictly approaching pseudo-lines and a pseudo-line $\ell \in A$, any vertical translation of $\ell$ in *A* results again in an arrangement of strictly approaching pseudo-lines.

Doing an arbitrary translation, we may run into trouble when the pseudo-lines are not strictly approaching. In this case it can happen that two pseudo-lines share an infinite number of points. The following lemma allows us replace non-strictly approaching arrangements by *x*-isomorphic strictly approaching arrangements.

### Lemma 2.4

Any simple arrangement of approaching pseudo-lines is homeomorphic to a polygonal *x*-isomorphic arrangement of strictly approaching pseudo-lines.

### Proof

Given an arrangement *A*, construct a polygonal arrangement $A'$ as described for Lemma 2.1. If the resulting pseudo-lines are strictly approaching, we are done. Otherwise, consider the rays that emanate to the left. We may change their slopes such that all the slopes are different and their relative order remains the same. Consider the first vertical slab defined by two neighboring vertical lines *v* and *w* that contains two segments that are parallel (if there are none, the arrangement is strictly approaching). Choose a vertical line $v'$ slightly to the left of the slab and use $v'$ and *w* as helper-lines to redraw the pseudo-lines in the slab. Since the arrangement is simple the resulting arrangement is *x*-isomorphic and it has fewer parallel segments. Iterating this process yields the desired result. $\square$

### Lemma 2.5

If *A* is an approaching arrangement with a non-simple allowable sequence, then there exists an approaching arrangement $A'$ whose allowable sequence is a refinement of the allowable sequence of *A*, i.e., the sequence of $A'$ may have additional permutations between consecutive pairs $\pi ,\pi '$ in the sequence of *A*.

### Proof

Since its allowable sequence is non-simple, arrangement *A* has a crossing point where more than two pseudo-lines cross or *A* has several crossings with the same *x*-coordinate. Let $\ell$ be a pseudo-line participating in such a degeneracy. Translating $\ell$ slightly in vertical direction a degeneracy is removed and the allowable sequence is refined. $\square$

Ringel’s homotopy theorem [4, Thm. 6.4.1] tells us that given a pair *A*, *B* of pseudo-line arrangements, *A* can be transformed to *B* by homeomorphisms of the plane and so-called *triangle flips*, where a pseudo-line is moved over a crossing. Within the subset of arrangements of approaching pseudo-lines, the result still holds. We first show a specialization of Ringel’s isotopy result [4, Prop. 6.4.2]:

### Lemma 2.6

Two *x*-isomorphic arrangements of approaching pseudo-lines can be transformed into each other by a homeomorphism of the plane such that all intermediate arrangements are *x*-isomorphic and approaching.

### Proof

Given an arrangement *A* of approaching pseudo-lines, we construct a corresponding polygonal arrangement $A'$. Linearly transforming a point $f_i(x)$ on a pseudo-line $\ell _i$ in *A* to the point $f'_i(x)$ on the corresponding line $\ell '_i$ in $A'$ gives a homeomorphism from *A* to $A'$ which can be extended to the plane. Given two *x*-isomorphic arrangements $A'$ and *B* of polygonal approaching pseudo-lines, we may shift helper-lines horizontally, so that the $$\left( {\begin{array}{c}n\\ 2\end{array}}\right) +1$$ helper-lines of the two arrangements become adjusted, i.e., are at the same *x*-coordinates; again there is a corresponding homeomorphism of the plane. Now recall that these arrangements can be obtained from solutions of linear programs. Since $A'$ and *B* have the same combinatorial structure, their defining inequalities are the same. Thus, a convex combination of the variables defining the two arrangements is also in the solution space, which continuously takes us from $A'$ to *B* and thus completes the proof. $\square$

### Theorem 2.7

Given two simple arrangements of approaching pseudo-lines, one can be transformed to the other by homeomorphisms of the plane and triangle flips such that all intermediate arrangement are approaching.

### Proof

Let $A_0$ be a fixed simple arrangement of *n* lines. We show that any approaching arrangement *A* can be transformed into $A_0$ with the given operations. Since the operations are invertible this is enough to prove that if (*A*, *B*) is a pair of approaching arrangements, then *A* can be transformed into *B* with the given operations.

**Fig. 3:**
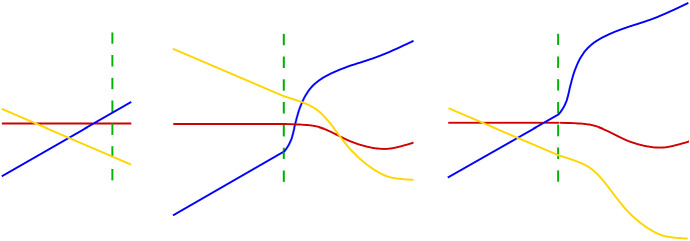
A line arrangement $A_0$ (left) and the arrangements $A'$ and $A''$ used for the transformation from *A* to $A_0$

Consider a vertical line $\ell$ in *A* such that all the crossings of *A* are to the right of $\ell$ and replace the part of the pseudo-lines of *A* left of $\ell$ by rays with the slopes of the lines of $A_0$. This yields an arrangement $A'$ isomorphic to *A*, see Fig. 3. This replacement is covered by Lemma 2.6. Let $\ell _0$ be a vertical line in $A_0$ which has all the crossings of $A_0$ to the left. Now we vertically shift the pseudo-lines of $A'$ to make their intersections with $\ell$ an identical copy of their intersections with $\ell _0$. This yields an arrangement $A''$ isomorphic to $A_0$, see Fig. 3. During the shifting we have a continuous family of approaching arrangements which can be described by homeomorphisms of the plane and triangle flips. To get from $A''$ to $A_0$ we only have to replace the part of the pseudo-lines of *A* to the right of $\ell$, where no crossings remain, by rays which have the same slopes of the lines of $A_0$. This makes all the pseudo-lines actual lines and the arrangement is identical to $A_0$. $\square$

Note that the proof requires the arrangement to be simple. Vertical translations of pseudo-lines now allows us to prove a restriction of our motivating question.

### Theorem 2.8

An arrangement of approaching red and blue pseudo-lines contains a triangular cell that is bounded by both a red and a blue pseudo-line unless it is a pencil, i.e., all the pseudo-lines cross in a single point.

### Proof

By symmetry in color and direction we may assume that there is a crossing of two blue pseudo-lines above a red pseudo-line. Translate all the red pseudo-lines upwards with the same speed. Consider the first moment $t>0$ when the isomorphism class changes. This happens when a red pseudo-line moves over a blue crossing, or a red crossing is moved over a blue pseudo-line. In both cases the three pseudo-lines have determined a bichromatic triangular cell of the original arrangement.

Now consider the case that at time *t* parallel segments of different color are concurrent. In this case we argue as follows. Consider the situation at time $\varepsilon >0$ right after the start of the motion. Now every multiple crossing is monochromatic and we can use an argument as in the proof of Lemma 2.4 to get rid of parallel segments of different colors. Continuing the translation after the modification reveals a bichromatic triangle as before. $\square$

## Levi’s Lemma for Approaching Arrangements

Proofs for showing that well-known properties of line arrangements generalize to pseudo-line arrangements often use Levi’s enlargement lemma. (For example, Goodman and Pollack [9] give generalizations of Radon’s theorem, Helly’s theorem, etc.) Levi’s lemma states that a pseudo-line arrangement can be augmented by a pseudo-line through any pair of points. In this section, we show that we can add a pseudo-line while maintaining the property that all pseudo-lines of the arrangement are approaching.

### Lemma 3.1

Given an arrangement of approaching pseudo-lines containing two pseudo-lines $l_i$ and $l_{i+1}$ (each a function $\mathbb {R} \rightarrow \mathbb {R}$), consider $l' = l'(x) = \lambda l_i(x) + (1-\lambda ) l_{i+1}(x)$, for some $0 \le \lambda \le 1$. The arrangement augmented by $l'$ is still an arrangement of approaching pseudo-lines.

### Proof

Consider any pseudo-line $l_j$ of the arrangement, $j \!\le \! i$. We know that for $x_1\!<\!x_2$, $l_j(x_1) - l_i(x_1) \ge l_j(x_2) - l_i(x_2)$, whence $\lambda l_j(x_1) - \lambda l_i(x_1) \ge \lambda l_j(x_2) - \lambda l_i(x_2)$. Similarly, we have $(1-\lambda ) l_j(x_1) - (1-\lambda ) l_{i+1}(x_1)\ge (1-\lambda ) l_j(x_2) - (1-\lambda ) l_{i+1}(x_2)$. Adding these two inequalities, we get

$$\begin{aligned} l_j(x_1) - l'(x_1) \ge l_j(x_2) -l'(x_2) . \end{aligned}$$

The analogous holds for any $j \ge i+1$. $\square$

The lemma gives us a means of producing a convex combination of two approaching pseudo-lines with adjacent slopes. Note that the adjacency of the slopes was necessary in the above proof.

### Lemma 3.2

Given an arrangement of *n* approaching pseudo-lines, we can add a pseudo-line $l_{n+1} = l_{n+1}(x) = l_n(x) + \delta (l_{n}(x) - l_{n-1}(x))$ for any $\delta > 0$ and still have an approaching arrangement.

### Proof

Assuming $$x_2 > x_1$$ implies

$$\begin{aligned}&l_n(x_1) - l_{n+1}(x_1) = l_n(x_1) - l_n(x) - \delta (l_{n}(x_1) - l_{n-1}(x_1)) = \delta (l_{n-1}(x_1) - l_{n}(x_1))\\&\qquad \qquad \qquad \qquad \quad \ge \delta (l_{n-1}(x_2) - l_{n}(x_2)) = l_n(x_2) - l_{n+1}(x_2). \end{aligned}$$

With $l_j(x_1) - l_{n}(x_1) \ge l_j(x_2) - l_{n}(x_2)$ we also get $l_j(x_1) - l_{n+1}(x_1) \ge l_j(x_2) - l_{n+1}(x_2)$ for all $1\le j < n$. $\square$

### Theorem 3.3

Given an arrangement of strictly approaching pseudo-lines and two points *p* and *q* with different *x*-coordinates, the arrangement can be augmented by a pseudo-line $l'$ containing *p* and *q* to an arrangement of approaching pseudo-lines. Further, if *p* and *q* do not have the same vertical distance to a pseudo-line of the initial arrangement, then the resulting arrangement is strictly approaching.

### Proof

Let *p* have smaller *x*-coordinate than *q*. Vertically translate all pseudo-lines such that they pass through *p* (the pseudo-lines remain strictly approaching, forming a pencil through *p*). If there is a pseudo-line that also passes through *q*, we add a copy $l'$ of it. If *q* is between $l_i$ and $l_{i+1}$, then we find some $0<\lambda <1$ such that $l'(x)=\lambda l_i(x) + (1-\lambda ) l_{i+1}(x)$ contains *p* and *q*. By Lemma 3.1 we can add $l'$ to the arrangement. If *q* is above or below all pseudo-lines in the arrangement, we can use Lemma 3.2 to add a pseudo-line; we choose $\delta$ large enough such that the new pseudo-line contains *q*. Finally translate all pseudo-lines back to their initial position. This yields an approaching extension of the original arrangement with a pseudo-line containing *p* and *q*. Observe that the arrangement is strictly approaching unless the new pseudo-line was chosen as a copy of $l'$. $\square$

Following Goodman et al. [14], a *spread of pseudo-lines* in the Euclidean plane is an infinite family of simple curves such that

(i) each curve is asymptotic to some line at both ends,
(ii) every two curves intersect at one point, at which they cross, and
(iii) there is a bijection *L* from the unit circle *C* to the family of curves such that *L*(*p*) is a continuous function (under the Hausdorff metric) of $p \in C$.

It is known that every projective arrangement of pseudo-lines can be extended to a spread [14] (see also [13]). For Euclidean arrangements this is not true because condition (i) may fail (for an example take the parabolas $(x-i)^2$ as pseudo-lines). However, given an Euclidean arrangement *A* we can choose two vertical lines $v_-$ and $v_+$ such that all the crossings are between $v_-$ and $v_+$ and replace the extensions beyond the vertical lines by appropriate rays. The result of this procedure is called the *truncation* of *A*. Note that the truncation of *A* and *A* are *x*-isomorphic and if *A* is approaching then so is the truncation. We use Lemma 3.1 to show the following.

### Theorem 3.4

The truncation of every approaching arrangement of pseudo-lines can be extended to a spread of pseudo-lines and a single vertical line such that the non-vertical pseudo-lines of that spread are approaching.

### Proof

Let $l_1, \ldots , l_n$ be the pseudo-lines of the truncation of an approaching arrangement. Add two almost vertical straight lines $l_0$ and $l_{n+1}$ such that the slope of the line connecting two points on a pseudo-line $l_i$ is between the slopes of $l_0$ and $l_{n+1}$. The arrangement with pseudo-lines $l_0,l_1, \ldots , l_n,l_{n+1}$ is still approaching. Initialize *S* with these $n+2$ pseudo-lines. For each $0\le i \le n$ and each $\lambda \in (0,1)$ add the pseudo-line $\lambda l_i(x) + (1-\lambda ) l_{i+1}(x)$ to *S*. The proof of Lemma 3.1 implies that any two pseudo-lines in *S* are approaching. Finally, let *p* be the intersection point of $l_0$ and $l_{n+1}$, and add all the lines containing *p* and some point above these two lines to *S*. This completes the construction of the spread *S*. $\square$

## Approaching Generalized Configurations

Levi’s lemma is the workhorse in the proofs of many properties of pseudo-line arrangements. Among these, there is the so-called *double dualization* by Goodman and Pollack [10] that creates, for any arrangement of pseudo-lines, a corresponding primal generalized configuration of points.

Let us briefly recall their terminology. A *generalized configuration of points* is an arrangement of pseudo-lines with a specified set of *n* vertices, called *points*, such that any pseudo-line passes through two points, and, at each point, $n-1$ pseudo-lines cross. We assume for simplicity that there are no other vertices in which more than two pseudo-lines of the arrangement cross.

Let $\mathcal {C} = (\mathcal {A}, P)$ be a generalized configuration of points consisting of an approaching arrangement $\mathcal {A}$, and a set of points $P = \{p_1, \ldots , p_n\}$, which are labeled by increasing *x*-coordinate. We denote the pseudo-line of $\mathcal {A}$ connecting points $p_i, p_j \in P$ by $p_{ij}$.

Consider a point moving from top to bottom at left infinity. This point traverses all the pseudo-lines of $\mathcal {A}$ in some order. We claim that if we start at the top with the identity permutation $\pi = (1, \ldots , n)$, then, when passing $p_{ij}$ we can apply the (adjacent) transposition (*i*, *j*) to $\pi$. Moreover, by recording all the permutations generated during the move of the point we obtain an allowable sequence $\Pi _{\mathcal {C}}$.

Consider the complete graph $K_P$ on the set *P*. Let *c* be an unbounded cell of the arrangement $\mathcal {A}$, when choosing *c* as the north-face of $\mathcal {A}$ we get a left to right orientation on each $p_{ij}$. Let this induce the orientation of the edge $\{i,j\}$ of $K_P$. These orientations constitute a tournament on *P*. It is easy to verify that this tournament is acyclic, i.e., it induces a permutation $\pi _c$ on *P*. The following can be derived from these definitions, see [10] for more details.

- The order $\pi _0$ corresponding to the top cell equals the left-to-right order on *P*. Since we have labeled the points by increasing *x*-coordinate this is the identity.
- When traversing $p_{ij}$ to get from a cell *c* to an adjacent cell $c'$ the two orientations of the complete graph only differ in the orientation of the edge $\{i,j\}$. Hence, $\pi _c$ and $\pi _{c'}$ are related by the adjacent transposition (*i*, *j*).

Note that the allowable sequence $\Pi _{\mathcal {C}}$ and the allowable sequence of $\mathcal {A}$ are different objects, they differ even in the length of the permutations.

We say that an arrangement of pseudo-lines is *dual* to a (*primal*) generalized configuration of points if they have the same allowable sequence. Goodman and Pollack [10] showed that for every pseudo-line arrangement there is a primal generalized configuration of points, and vice versa. We prove the same for the sub-class of approaching arrangements.

### Lemma 4.1

For every generalized configuration $\mathcal {C} \!=\! (\mathcal {A}, P)$ of points on an approaching arrangement $\mathcal {A}$, there is an approaching arrangement ${A}^*$ with allowable sequence $\Pi _{\mathcal {C}}$.

**Fig. 4:**
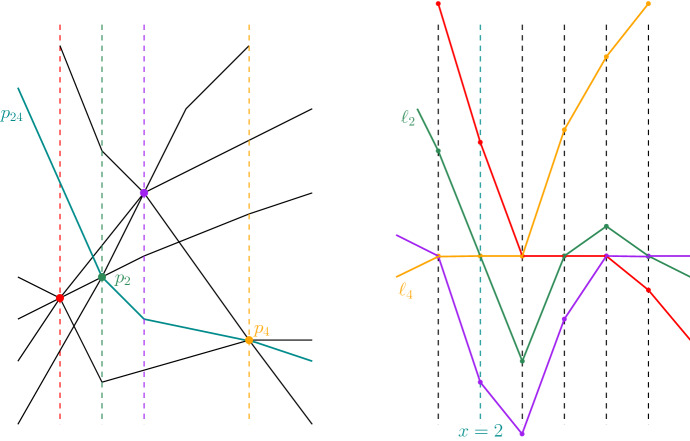
An approaching generalized configuration (left) and its dual approaching arrangement (right)

For an illustration of the construction, see Fig. 4.

### Proof

Let $\Pi _{\mathcal {C}} = \pi _0,\pi _1,\ldots ,\pi _h$. We call (*i*, *j*) the adjacent *transposition at g* when $\pi _g=(i,j)\circ \pi _{g-1}$. To produce a polygonal approaching arrangement $A^*$ we define the *y*-coordinates of the pseudo-lines $\ell _1,\ldots ,\ell _n$ at *x*-coordinates $i\in [h]$. Let (*i*, *j*) be the transposition at *g*. Consider the pseudo-line $p_{ij}$ of $\mathcal {C}$. Since $p_{ij}$ is *x*-monotone we can evaluate $p_{ij}(x)$. The *y*-coordinate of the pseudo-line $\ell _k$ dual to the point $p_k=(x_k,y_k)$ at $x=g$ is obtained as $y_{k}(g)=p_{ij}(x_k)-p_k(x_k)$, i.e., the vertical distance between the pseudo-line $p_{ij}$ and the point $p_k$. We then extend $\ell _k$ linearly between the defined points.

We argue that the resulting pseudo-line arrangement is approaching. Let (*i*, *j*) and (*s*, *t*) be transpositions at *g* and $g'$, respectively, and assume $g < g'$. We have to show that $y_{a}(g) - y_{b}(g) \ge y_{a}(g') - y_{b}(g')$, for all $1\le a < b \le n$. From $a < b$ it follows that $p_a$ is left of $p_b$, i.e., $x_a < x_b$. The pseudo-lines $p_{ij}$ and $p_{st}$ are approaching, hence $p_{ij}(x_a) - p_{st}(x_a) \ge p_{ij}(x_b) - p_{st}(x_b)$, i.e., $p_{ij}(x_a) - p_{ij}(x_b) \ge p_{st}(x_a) - p_{st}(x_b)$, which translates to $y_{a}(g) - y_{b}(g) \ge y_{a}(g')-y_{b}(g')$.

Further, it follows from the construction that for for the transposition (*i*, *j*) at *g*, the constructed dual pseudo-lines intersect at $x_g$. In particular, this implies that the allowable sequence of ${A}^*$ equals $\Pi _{\mathcal {C}}$. This completes the proof. $\square$

Goodman and Pollack use the so-called *double dualization* to show how to obtain a primal generalized configuration of points for a given arrangement *A* of pseudo-lines. In this process, they add a pseudo-line through each pair of crossings in *A*, using Levi’s enlargement lemma. This results in a generalized configuration $\mathcal {C}'$ of points, where the points are the crossings of *A*. From this, they produce the dual pseudo-line arrangement $\mathcal {A}'$. Then, they repeat the previous process for $\mathcal {A}'$ (that is, adding a line through all pairs of crossings of $\mathcal {A}'$). The result is a generalized configuration $\mathcal {C}$ of points, which they show being the primal generalized configuration of $\mathcal {A}$. With Theorem 3.3 and Lemma 4.1, we know that both the augmentation through pairs of crossings and the dualization process can be done such that we again have approaching arrangements, yielding the following result.

### Lemma 4.2

For every arrangement of approaching pseudo-lines, there is a primal generalized configuration of points whose arrangement is also approaching.

Combining Lemmas 4.1 and 4.2, we obtain the main result of this section.

### Theorem 4.3

An allowable sequence is the allowable sequence of an approaching generalized configuration of points if and only if it is the allowable sequence of an approaching arrangement.

## Realizability and Counting

Considering the freedom one has in constructing approaching arrangements, one may wonder whether actually all pseudo-line arrangements are *x*-isomorphic to approaching arrangements. As we will see in this section, this is not the case. We use the following lemma, that can easily be shown using the construction from the proof of Lemma 2.1.

### Lemma 5.1

Given a simple suballowable sequence of permutations $(\mathrm{id}, \pi _1, \pi _2)$, where $\mathrm{id}$ is the identity permutation, the suballowable sequence is realizable with an arrangement of approaching pseudo-lines if and only if it is realizable as a line arrangement.

### Proof

Consider any realization *A* of the simple suballowable sequence with an arrangement of approaching pseudo-lines. Since the arrangement is simple, we can consider the pseudo-lines as being strictly approaching, due to Lemma 2.4. There exist two vertical lines $v_1$ and $v_2$ such that the order of intersections of the pseudo-lines with them corresponds to $\pi _1$ and $\pi _2$, respectively. We claim that replacing pseudo-line $p_i\in A$ by the line $\ell _i$ connecting the points $(v_1,p_i(v_1))$ and $(v_2,p_i(v_2))$ we obtain a line arrangement representing the suballowable sequence $(\mathrm{id}, \pi _1, \pi _2)$.

To prove the claim we verify that for $i < j$ the slope of $\ell _i$ is less than the slope of $\ell _j$. Since *A* is approaching we have $p_i(v_1) - p_j(v_1) \ge p_i(v_2) - p_j(v_2)$, i.e., $p_i(v_1) - p_i(v_2) \ge p_j(v_1) - p_j(v_2)$. The slopes of $\ell _i$ and $\ell _j$ are obtained by dividing both sides of this inequality by $v_1-v_2$, which is negative. $\square$

Asinowski [3] identified a suballowable sequence $(\mathrm{id}, \pi _1, \pi _2)$, with permutations of six elements which is not realizable with an arrangement of lines.

### Corollary 5.2

There exist simple suballowable sequences that are not realizable by arrangements of approaching pseudo-lines.

**Fig. 5:**
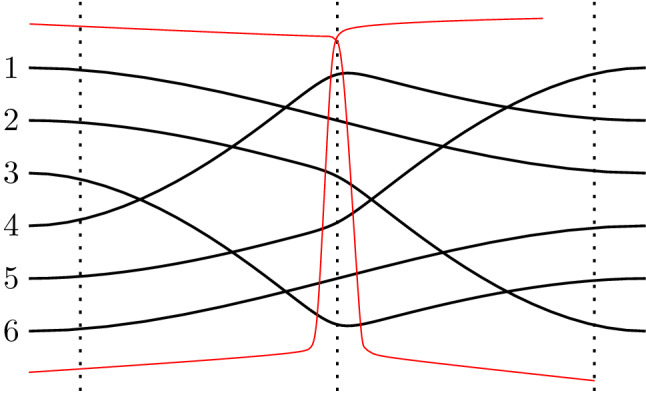
A part of a six-element pseudo-line arrangement (bold) whose suballowable sequence (indicated by the vertical lines) is non-realizable (adapted from [3, Fig. 4]). Adding the two thin pseudo-lines crossing in the vicinity of the vertical line crossed by the pseudo-lines in the order of $\pi _1$ and doing the same for $\pi _2$ enforces that the allowable sequence of any isomorphic arrangement contains the subsequence $(\mathrm{id}, \pi _1, \pi _2)$

With the modification of Asinowski’s example shown in Fig. 5, we obtain an arrangement not having an isomorphic approaching arrangement. The modification adds two almost-vertical lines crossing in the north-cell such that they form a wedge crossed by the lines of Asinowski’s example in the order of $\pi _1$. We do the same for $\pi _2$. The resulting object is a simple pseudo-line arrangement, and each isomorphic arrangement contains Asinowski’s sequence.

### Corollary 5.3

There are pseudo-line arrangements for which there exists no isomorphic arrangement of approaching pseudo-lines.

Aichholzer et al. [1] construct a suballowable sequence $(\mathrm{id}, \pi _1, \pi _2)$ on *n* lines such that all line arrangements realizing them require slope values that are exponential in the number of lines. Thus, also vertex coordinates in a polygonal representation as an approaching arrangement are exponential in *n*.

Ringel’s Non-Pappus arrangement [20] shows that there are allowable sequences that are not realizable by straight lines. It is not hard to show that the Non-Pappus arrangement has a realization with approaching pseudo-lines. We will show that in fact the number of approaching arrangements, is asymptotically larger than the number of arrangements of lines.

### Theorem 5.4

There exist $2^{\Theta (n^2)}$ isomorphism classes of simple arrangements of *n* approaching pseudo-lines.

### Proof

**Fig. 6:**
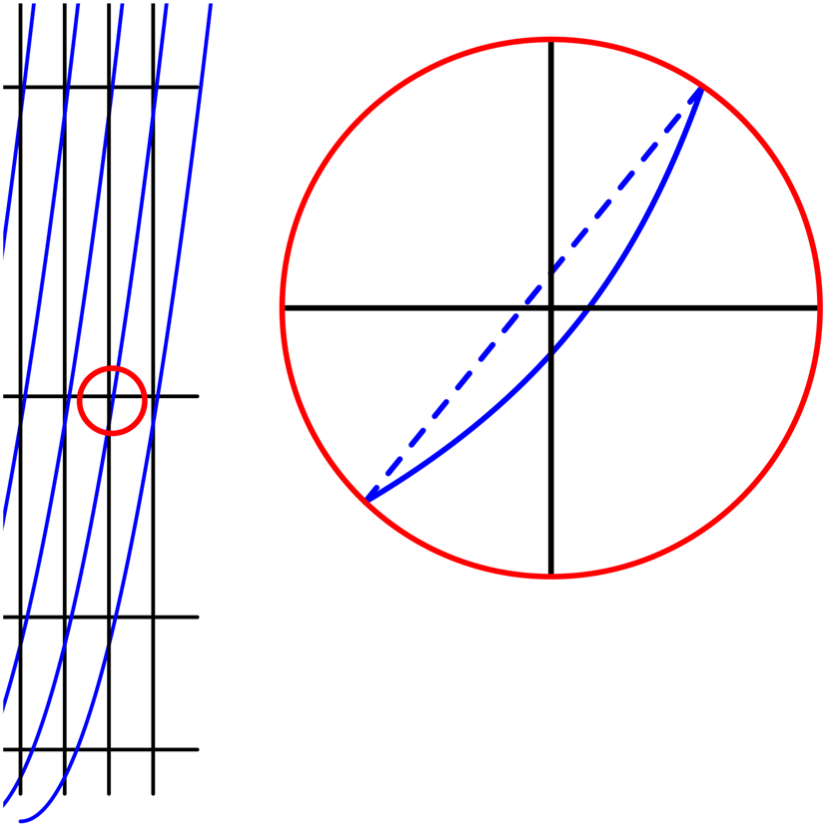
A construction for an $2^{\Omega (n^2)}$ lower bound on the isomorphism classes of approaching arrangements

The upper bound follows from the number of non-isomorphic arrangements of pseudo-lines. Our lower-bound construction is an adaptation of the construction presented by Matoušek [17, p. 134] for general pseudo-line arrangements. See the left part of Fig. 6 for a sketch of the construction. We start with a construction containing parallel lines that we will later perturb. Consider a set *V* of vertical lines $v_i : x = i$, for $i\in [n]$. Add horizontal pseudo-lines $h_i:y=i^2$, for $i\in [n]$. Finally, add parabolic curves $p_i:y=(x+i)^2-\varepsilon$, defined for $x \ge 0$, some $0<\varepsilon \ll 1$, and $i \in [n]$ (we will add the missing part towards left infinity later). Now, $p_i$ passes slightly below the crossing of $h_{i+j}$ and $v_j$ at $(j,(i+j)^2)$. See the left part of Fig. 6 for a sketch of the construction. We may modify $p_i$ to pass above the crossing at $(j,(i+j)^2)$ by replacing a piece of the curve near this point by a line segment with slope $2(i+j)$; see the right part of Fig. 6. Since the derivatives of the parabolas are increasing and the derivatives of $p_{i+1}$ at $j - 1$ and of $p_{i-1}$ at $j + 1$ are both $2(j+i)$ the vertical distances from the modified $p_i$ to $p_{i+1}$ and $p_{i-1}$ remain increasing, i.e., the arrangement remains approaching.

For each crossing $(j,(i+j)^2)$, we may now independently decide whether we want $p_i$ to pass above or below the crossing. The resulting arrangement contains parallel and vertical lines, but no three points pass through a crossing. This means that we can slightly perturb the horizontal and vertical lines such that the crossings of a horizontal and a vertical remain in the vicinity of the original crossings, but no two lines are parallel, and no line is vertical. To finish the construction, we add rays from the points on $p_i$ with $x=0$, each having the slope of $p_i$ at $x=0$. Each arrangement of the resulting class of arrangements is approaching. We have $\Theta (n^2)$ crossings for which we make independent binary decisions. Hence the class consists of $2^{\Theta (n^2)}$ approaching arrangements of 3*n* pseudo-lines. $\square$

As there are only $2^{\Theta (n \log n)}$ isomorphism classes of simple line arrangements [12], we see that we have way more arrangements of approaching pseudo-lines.

The number of allowable sequences is $2^{\Theta (n^2 \log n)}$ [21]. We show next that despite of the existence of nonrealizable suballowable sequences (Corollary 5.2), the number of allowable sequences for approaching arrangements, i.e., the number of *x*-isomorphism classes of these arrangements, is asymptotically the same as the number of all allowable sequences.

### Theorem 5.5

There are $2^{\Theta (n^2 \log n)}$ allowable sequences realizable as arrangements of approaching pseudo-lines.

### Proof

The upper bound follows from the number of allowable sequences. For the lower bound, we use the construction in the proof of Theorem 5.4, but omit the vertical lines. Hence, we have the horizontal pseudo-lines $h_i : y = i^2$ and the paraboloid curves $p_i : y = (x + i)^2-\varepsilon$, defined for $x\ge 0$ and $0 < \varepsilon \ll 1$. For a parabolic curve $p_i$ and a horizontal line $h_{i+j}$, consider the neighborhood of the point $(j,(i+j)^2)$. Given a small value $\alpha$ we can replace a piece of $p_i$ by the appropriate line segment of slope $2(i+j)$ such that the crossing of $h_{i+j}$ and the modified $p_i$ has *x*-coordinate $j-\alpha$.

For fixed *j* and any permutation $\pi$ of $[n-j]$ we can define values $\alpha _i$ for $i \in [n-j]$ such that $\alpha _{\pi (1)}< \alpha _{\pi (2)}<\ldots <\alpha _{\pi (n-j)}$. Choosing the offset values $\alpha _i$ according to different permutations $\pi$ yields different vertical permutations in the neighborhood of $x=j$, i.e., the allowable sequences of the arrangements differ. Hence, the number allowable sequences of approaching arrangements is at least the superfactorial $\prod _{j=1}^{n} j!$, which is in $2^{\Omega (n^2 \log n)}$. $\square$

We have seen that some properties of arrangements of lines are inherited by approaching arrangements. It is known that every simple arrangement of pseudo-lines has $n-2$ triangles, the same is true for non-simple non-trivial arrangements of lines, however, there are non-simple non-trivial arrangements of pseudo-lines with fewer triangles, see [7]. We conjecture that in this context approaching arrangements behave like line arrangements.

### Conjecture 5.6

Every non-trivial arrangement of *n* approaching pseudo-lines has at least $n-2$ triangles.

## Approaching Arrangements in 3D

We have seen that approaching arrangements of pseudo-lines form an interesting class of arrangements of pseudo-lines. In this section we study the 3-dimensional version, this requires quite some technicalities. Therefore, before entering the detailed treatment of the subject we give an informal description of the results.

We consider pseudo-planes as functions $f:\mathbb {R}^2\rightarrow \mathbb {R}$. An arrangement of pseudo-planes is *approaching* if we can shift the pseudo-planes up and down independently and maintain the property that they form an arrangement.

Consider an arrangement of approaching pseudo-lines $f_1,f_2,\ldots ,f_n$ with the property that for all $x\in \mathbb {R}$ the slopes of all $f_i$ at *x* are uniquely defined. Considering the slopes of the pseudo-lines over any point *x* we have $s_1\le s_2\le \ldots \le s_n$, i.e., the point $(s_1, \ldots ,s_n)$ is in the closure of the set of points with $s_1<s_2<\ldots <s_n$. A vector of reals can be interpreted as a labeled Euclidean order type in one dimension. Slope vectors of approaching arrangements are in the closure of all vectors whose order type is the same as the order type of $(1,2,\ldots ,n)$. We can sloppily state this as: The order of slopes is in the closure of the order type of the identity permutation.

In the case of arrangements of pseudo-planes we can talk about the gradients over a point (*x*, *y*). A set $p_1,p_2,\ldots ,p_k$ of gradients can equivalently be viewed as a labeled set of points in the plane. This set of points defines an order type. It turns out that an approaching arrangement of pseudo-planes can be characterized by a non-degenerate order type $\chi$ in the sense that for every point (*x*, *y*) in the plane the order type of the gradients over this point is in the closure of $\chi$. This ends the informal part.

An *arrangement of pseudo-planes* in $\mathbb {R}^3$ is a finite set *A* of graphs of functions $f_i:\mathbb {R}^2\rightarrow \mathbb {R}$ such that the projection of all points of $\mathbb {R}^3$ that belong to at least two of the function graphs to the plane $\mathbb {R}^2\times \{0\}$ is an arrangement of pseudo-lines. In particular the intersection of any two of the functions projects to a pseudo-line and the intersection of any three of them is a point.

We define arrangements of approaching pseudo-planes via one of the key properties observed for arrangements of approaching pseudo-lines (Observation 2.3).

An *arrangement of approaching pseudo-planes* in $\mathbb {R}^3$ is an arrangement of pseudo-planes $h_1,\ldots ,h_n$ where each pseudo-plane $h_i$ is the graph of a smooth function $f_i:\mathbb {R}^2\rightarrow \mathbb {R}$ such that for any $c_1,\ldots ,c_n\in \mathbb {R}$, the graphs of $f_1+c_1,\ldots , f_n+c_n$ form a valid arrangement of pseudo-planes. This means that we can move the pseudo-planes up and down along the *z*-axis while maintaining the properties of a pseudo-plane arrangement. Clearly, arrangements of planes (no two of them parallel) are approaching.

In the following, we will assume that all functions $f_i$ are *Morse functions*, that is, they have no degenerate critical points.^4^ This is not a strong assumption as Morse functions are an open and dense subset of all smooth functions.

Let *G* be a collection of graphs of Morse functions $f_i:\mathbb {R}^2\rightarrow \mathbb {R}$. For any point (*x*, *y*) in $\mathbb {R}^2$, which we call the *underlying plane*, let $p_i(x,y)$ be the gradient of $f_i$ above (*x*, *y*). We may consider the gradients as points $p_i(x,y)$ in a plane, called the *plane attached at (x, y)*. We call $p_i(x,y)$ a *characteristic point* and let $P_G(x,y)$ be the set of characteristic points in the plane attached at (*x*, *y*). The Euclidean order type of the point set $P_G(x,y)$ is the *characteristic order type* of *G* at (*x*, *y*), it is denoted $\chi _G(x,y)$.

We denote by $p_i$ the set of characteristic points $p_i(x,y)$, taken over the entire underlying plane. Note that for every graph in an arrangement of approaching pseudo-planes, the characteristic points define a continuous vector field $p_i:\mathbb {R}^2\rightarrow \mathbb {R}^2$, namely its gradient vector field. We call $p_i$ a *characteristic field*. We denote by $P_G$ the set of characteristic fields $p_i$. Similarly, we denote by $\chi _G$ the set of characteristic order types of *G* on the whole underlying plane, that is, $\chi _G=\{\chi _G(x,y)\mid (x,y)\in \mathbb {R}^2\}$. We say that $\chi _G$ is *admissible* if the following conditions hold:

- for any pair $p_1,p_2$ of characteristic fields, the vector field $p_2-p_1$ has no critical points;
- for any two points $(x_1,y_1)$ and $(x_2,y_2)$ in the underlying plane, we have that if an ordered triple of characteristic points in $P_G(x_1, y_1)$ is positively oriented, then the corresponding triple in $P_G(x_2,y_2)$ is either positively oriented or collinear;
- for any triple $p_1,p_2,p_3$ of characteristic fields, the set of points in the underlying plane for which $p_1, p_2, p_3$ are collinear is either the whole plane or has no 2-dimensional components (i.e., for each (*x*, *y*) in this set and for every neighborhood *N* of (*x*, *y*), we have that *N* contains points which are not in the set).

The main result of this section is that a collection of graphs of functions is an approaching arrangement of pseudo-planes if and only if its characteristic order type is admissible. Before we prove this, let us analyze the situation for for two functions respectively pseudo-planes $f_1,f_2:\mathbb {R}^2 \rightarrow \mathbb {R}$.

### Lemma 6.1

Let $f_1$ and $f_2$ be two Morse functions. Then the the graphs of $f_1+c_1$ and $f_2+c_2$ intersect in a pseudo-line for all $c_1,c_2\in \mathbb {R}$ if and only if the function $f_2-f_1$ is surjective and has no critical points.

In particular, condition (a) on admissible order types ensures that any two pseudo-planes are approaching.

### Proof

Note that being surjective is a necessary condition for the difference $f_2-f_1$, as otherwise we can translate them until they do not intersect. Thus, in the following, we will assume that $f_2$ is surjective.

First note that $f_1+c_1$ and $f_2+c_2$ intersect in a pseudo-line for all $c_1,c_2\in \mathbb {R}$ if and only if $g:=f_2-f_1$ intersects every plane $z=c$, for $c\in \mathbb {R}$ in a pseudo-line. In other words, we want that all the contour lines of the surjective function *g* are homeomorphic to lines. This holds if and only if *g* has no minima, maxima or saddles. On one hand, each minimum, maximum or saddle corresponds to a critical point. On the other hand, as there are no degenerate critical points by assumption, each critical point corresponds to a maximum, minimum or saddle. Thus, all contour lines of *g* are homeomorphic to lines if and only if *g* has no critical points. $\square$

We will also need the following technical lemma, which will help us finding contradictions to condition (c):

### Lemma 6.2

Let $p=(p_x,p_y)$ be a gradient field defined in some neighborhood *N* of (0, 0) and let $\beta$ be an *x*-monotone curve containing (0, 0) in its interior. Assume that $p_x(a,b)=0$ for every $(a,b)\in N$ on $\beta$. Then $p_x(a,b)=0$ for every $(a,b)\in N$.

### Proof

As $p=(p_x,0)+(0,p_y)$ is a gradient field if and only if both summands are gradient fields, it is enough to show the statement for the vector field $p':=(p_x,0)$. Assume for the sake of contradiction that $p'$ does not vanish everywhere in *N*. As it vanishes everywhere on the *x*-monotone curve $\beta$, the function $p_x$ depends on *y*, and in particular we have that ${dp_x}/{dy}$ is not the zero function on *N*. But then the curl of $p'$, which is computed as ${d}0/{dx}-{d}p_x/{dy}$ is not zero everywhere, implying that the vector field $p'$ has curl. The result now follows from a classic result from vector analysis: a vector field is a gradient vector field if and only if it has no curl (see, e.g., [16, Thm. 8.7]). In particular, $p'$ and thus also *p* cannot be gradient fields, which is a contradiction to our assumptions. $\square$

We are now ready to prove the main result of this section.

### Theorem 6.3

Let *G* be a collection of graphs of Morse functions $f_i:\mathbb {R}^2\rightarrow \mathbb {R}$. Then *G* is an arrangement of approaching pseudo-planes if and only if $\chi _G$ is admissible and all the differences between two functions are surjective.

### Proof

Again, being surjective is a necessary condition for the difference of two functions. Thus, in the following, we will assume that all the differences between two functions are surjective.

**Admissible** $\Rightarrow$ **approaching**   We first show that if $\chi _G$ is admissible then *G* is an arrangement of approaching pseudo-planes. Suppose *G* is not an arrangement of approaching pseudo-planes. From Lemma 6.1, we may assume that for any two functions $f_i$ and $f_j$ and any offsets $c_i,c_j$, the intersection of the graphs graphs of $f_i+c_i$ and $f_j+c_j$ projects to a pseudo-line. If $f_1+c_1,\ldots ,f_n+c_n$ violates being an arrangement, then there are three functions $f'_i=f_i+c_i$, $f'_j=f_j+c_j$, and $f'_k=f_k+c_k$ such that on $f'_i$, the two curves defined by the intersections with $f'_j$ and $f'_k$ do not project to an arrangement of two pseudo-lines. Hence, the two curves have multiple intersections, they touch at a point, or they intersect in an interval. The projections of intersections stay the same if we subtract $f'_i$ from all of them, i.e., we now consider $f''_i=0$, $f''_j=f'_j-f'_i$, and $f''_k=f'_k-f'_i$. Note that the gradient of $f''_i$ is 0 everywhere. If the union of the two curves $\gamma _j={f''_j}^{-1}(0)$ and $\gamma _k={f''_k}^{-1}(0)$ encloses a bounded region, then the intersection of $f''_j$ and $f''_k$ attains an extremum in this region. With a translation of the original functions we can get this extremum as a touching point of the curves.

**Fig. 7:**
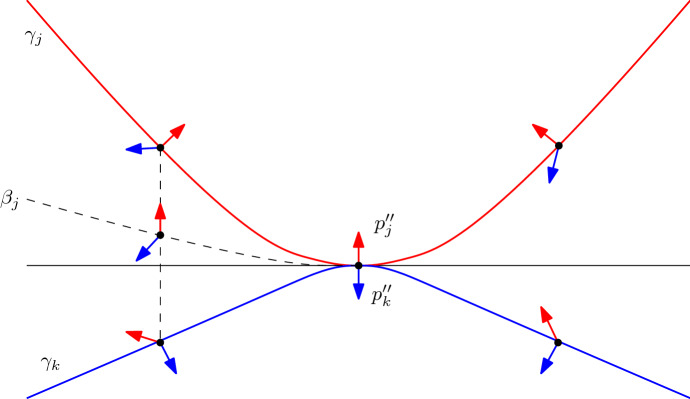
An illustration of a neighborhood of a touching point

For an illustration of the following arguments, see Fig. 7. Without loss of generality, we make the following assumptions:

- The touching point of $\gamma _j$ and $\gamma _k$ is (0, 0);
- the *x*-axis is tangent to $\gamma _j$ at this point, and as the two curves touch, the *x*-axis is also tangent to $\gamma _k$;
- $\gamma _j$ lies above the *x*-axis and $\gamma _k$ lies below;
- the gradients of $f''_j$ and $f''_k$ at (0, 0) point upwards and downwards, respectively.

In other words, as at any point on one of the curves the gradient is orthogonal to the tangent to the curve, we have $p''_j(0,0)=(0,b_j)$ and $p''_k(0,0)=(0,-b_k)$, for $b_j,b_k>0$. Now, in any small enough neighborhood we have that the *y*-coordinates of $p''_j$ and $p''_k$ are positive and negative, respectively. Further, we have that for every (*a*, *b*) on $\gamma _j$, the *x*-coordinates of the gradient $p''_j$ are positive for $a<0$ and negative for $a>0$, and analogously for $\gamma _k$. Assume without loss of generality that the orientation of the gradients is always positive or 0, but never negative, as otherwise we get a contradiction to condition (b). Thus, at $(a,b)\in \gamma _k$, the *x*-coordinates of the gradient $p''_j$ are negative for $a<0$ and positive for $a>0$. By the mean value theorem there is thus an *x*-monotone curve $\beta _j$ on which the *x*-component of $p''_j$ vanishes. Hence, by Lemma 6.2, there is a neighborhood $N_j$ of (0, 0) in which the *x*-component of $p''_j$ vanishes. Analogously, there is a neighborhood $N_k$ of (0, 0) in which the *x*-component of $p''_k$ vanishes. But now the intersection of $N_j$ and $N_k$ is a neighborhood in which $p''_j$, $p''_k$, and 0 are collinear, which is a contradiction to condition (c).

It remains to show that $\gamma _j$ and $\gamma _k$ cannot intersect in an interval. For this, assume without loss of generality that the interval lies on the *x*-axis and contains (0, 0) in its interior. At any point on the interval, both gradients have *x*-coordinate 0, thus by Lemma 6.2, for both of them there is a neighborhood of (0, 0), in which their *x*-coordinate vanishes. In the intersection of these two neighborhoods, the two gradients are thus collinear with 0, which is again a contradiction to condition (c).

**Approaching** $\Rightarrow$ **admissible**   For the other direction consider an approaching arrangement of pseudo-planes and assume that $\chi _G$ is not admissible. We have already shown in Lemma 6.1 that if condition (a) is violated, then the two corresponding pseudo-planes are not approaching. So, assume now that condition (b) is violated, that is, there are three pseudo-planes $f_1,f_2,f_3$ whose characteristic fields $p_1,p_2,p_3$ change their orientation from positive to negative. In particular, they are collinear at some point. Assume without loss of generality that $f_2$ and $f_3$ are planes containing the origin whose characteristic fields are thus constant, and assume without loss of generality that they are $p_2=(0,1)$ and $p_3=(0,-1)$. In particular, the intersection of $f_2$ and $f_3$ is the *x*-axis in $\mathbb {R}^3$. Consider now a $\varepsilon$-disc *B* around the origin in $\mathbb {R}^2$ and let $B_<$, $B_0$, and $B_>$ be the subsets of *B* with $x<0$, $x=0$, and $x>0$, respectively. Assume without loss of generality that for $(x,y)\in B$ the characteristic point $p_1(x,y)$ is to the left of the *y*-axis in $B_<$, to the right in $B_>$, and on the *y*-axis in $B_0$. Also, assume that $f_1$ contains the origin in $\mathbb {R}^3$. But then, $f_1$ is below the (*x*, *y*)-plane everywhere in *B*. In particular, $f_1$ touches $f_2\cap f_3$ in a single point, namely the origin. Hence, $f_1\cap f_3$ and $f_2\cap f_3$ is not an arrangement of two pseudo-lines in $f_3$. Similar arguments show that if condition (c) is violated, then after some translation the intersection of some two pseudo-planes in a third one is an interval. $\square$

From the proof it is not evident to what extent an arrangement of approaching pseudo-planes is determined by its admissible family of characteristic order types. In particular, we would like to understand which admissible families of order types correspond to families of characteristic order types. A necessary condition follows again from the fact that a vector field is a gradient vector field of a scalar function if and only if it has no curl. This implies the following:

### Corollary 6.4

Let $(p_1,\ldots ,p_n)$ be a family of vector fields and for $(x,y)\in \mathbb {R}^2$ let *P*(*x*, *y*) be the order type of the points $p_1(x,y),\ldots ,p_n(x,y)$. Then $(p_1,\ldots ,p_n)$ is the characteristic field of an arrangement of approaching pseudo-planes if and only if

- $p_i$ is curl-free for each $i=1,\ldots ,n$ and
- $\{P(x,y)\mid (x,y)\in \mathbb {R}^2\}$ is an admissible collection of order types.

**Fig. 8:**
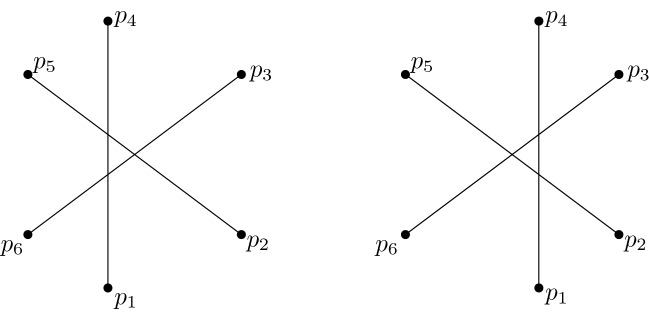
Two different arrangements induced by the same order type

Let $G=(g_1,\ldots ,g_n)$ be an arrangement of approaching pseudo-planes. A natural question is, whether *G* can be extended, that is, whether we can find a pseudo-plane $g_{n+1}$ such that $(g_1,\ldots ,g_n,g_{n+1})$ is again an arrangement of approaching pseudo-planes. Consider the realization of $\chi _G(x,y)$ for some $(x,y)\in \mathbb {R}^2$. Any two points in this realization span a line. Let $\mathcal {A}(x,y)$ be the line arrangement defined by all of these lines. Note that even if $\chi _G(x,y)$ is the same order type for every $(x,y)\in \mathbb {R}^2$, the realization might be different, i.e., $\mathcal {A}(x',y')$ and $\mathcal {A}(x,y)$ need not be isomorphic. For an illustration see Fig. 8. (This issue also comes up in the problem of extension of order types, e.g. in [19], where the authors count the number of order types with exactly one point in the interior of the convex hull.)

A cell of $\mathcal {A}(x,y)$ is defined by its sidedness with respect each line of $\mathcal {A}(x,y)$, i.e., to each pair $(p_i(x,y),p_j(x,y))$. A cell *c* of $\mathcal {A}(x,y)$ is *representable* in $\mathcal {A}(x',y')$ if there is a point *p* in $\mathcal {A}(x',y')$ which belongs to the same closed halfspaces as *c* in $\mathcal {A}(x,y)$. A *c* cell of $\mathcal {A}(x,y)$ is *admissible*, if it is representable in $\mathcal {A}(x',y')$ for every $(x',y')\in \mathbb {R}^2$. Clearly, if we can extend *G* with a pseudo-plane $g_{n+1}$, then its gradient, interpreted as a point *p* in the plane, must lie in an admissible cell *c*. On the other hand, as *c* is admissible, it is possible to move *p* continuously in *c*, and if all the characteristic fields $(p_1,\ldots ,p_n)$ are curl-free, then so is the vector field $p_{n+1}$ obtained this way. Thus, $p_{n+1}$ is the vector field of a function $f_{n+1}$ and by Corollary 6.4, its graph $g_{n+1}$ extends *G*. In particular, *G* can be extended if and only if $\mathcal {A}(x,y)$ contains an admissible cell. As the unbounded cells incident to an extremal characteristic point are always admissible, we get that every arrangement of approaching pseudo-planes can be extended. Furthermore, by the properties of approaching pseudo-planes, $g_{n+1}$ can be chosen to go through any given point *p* in $\mathbb {R}^3$. In conclusion, we get the following:

### Theorem 6.5

Let $G=(g_1,\ldots ,g_n)$ be an arrangement of approaching pseudo-planes and let *p* be a point in $\mathbb {R}^3$. Then there exists a pseudo-plane $g_{n+1}$ such that $(g_1,\ldots ,g_n,g_{n+1})$ is an arrangement of approaching pseudo-planes and *p* lies on $g_{n+1}$.

It is possible that only cells incident to a characteristic point are admissible. In such cases, every pseudo-plane $g_{n+1}$ that extends *G* is essentially a copy of one of the pseudo-planes of *G*. For some order types, there are cells that are not incident to a characteristic point but still appear in every possible realization, e.g. the unique pentagon defined by five points in convex position. It is an interesting open problem to characterize the cells which appear in every realization of an order type.

## Conclusion

In this paper, we introduced a class of pseudo-line arrangements that generalizes line arrangements, but still retains certain geometric properties. A major algorithmic open problems is to decide for a given pseudo-line arrangements whether it has a realization as an approaching arrangement. We also do not know how projective transformations influence this realizability. We hope that approaching arrangements continue to help bridge the gap between line arrangements and pseudo-line arrangements. We also have introduced approaching arrangements in three dimensions and expect that via the order type defined by normal vectors the concept can be lifted to higher dimensions.

## Footnotes

1 Pseudo-line arrangements are often studied in the real projective plane, with pseudo-lines being simple closed curves that do not separate the projective plane. All arrangements can be represented by *x*-monotone arrangements [10]. As *x*-monotonicity is crucial for our setting and the line at infinity plays a special role, we use the above definition.

2 This term will be defined in Sect. 2.

3 We do not get a polynomial algorithm by reading off the allowable sequence of the pseudo-line arrangement and then applying Theorem 2.2 as there are families of isomorphic pseudo-line arrangements with exponentially many different allowable sequences.

4 Recall that a critical point of a function $f:\mathbb {R}^2\rightarrow \mathbb {R}$ is a point where the gradient vanishes. A critical point is degenerate if the Hessian matrix (the matrix of second partial derivatives) is singular.
